# Molecular Mechanisms Involved in the Impairment of Boar Sperm Motility by Peroxynitrite-Induced Nitrosative Stress

**DOI:** 10.3390/ijms21041208

**Published:** 2020-02-11

**Authors:** Rebeca Serrano, Nicolás Garrido, Jose A. Céspedes, Lauro González-Fernández, Luis J. García-Marín, María J. Bragado

**Affiliations:** Research Group of Intracellular Signaling and Technology of Reproduction (Research Institute INBIO G+C), University of Extremadura, 10003 Cáceres, Spain; rebecasp@unex.es (R.S.); ngarridoff@unex.es (N.G.); jcespede@alumnos.unex.es (J.A.C.); lgonfer@unex.es (L.G.-F.); ljgarcia@unex.es (L.J.G.-M.)

**Keywords:** boar spermatozoa, motility, peroxynitrite, viability, lipid peroxidation, phosphorylation, PKA, GSK-3α

## Abstract

Excessive levels of reactive nitrogen species (RNS) produce nitrosative stress. Among RNS is peroxynitrite, a highly reactive free radical generated when nitric oxide reacts with superoxide anion. Peroxynitrite effects have been mainly studied in somatic cells, and in spermatozoa the majority of studies are focused in humans. The aim of this study is to investigate the in vitro peroxynitrite effect on boar spermatozoa functions and the molecular mechanisms involved. Spermatozoa were exposed to the donor 3-morpholinosydnonimine (SIN-1) in non-capacitating or capacitating medium, motility was evaluated by CASA, functional parameters by flow cytometry and sperm protein phosphorylation by Western blotting. SIN-1 treatment, that significantly increases peroxynitrite levels in boar spermatozoa, potentiates the capacitating-stimulated phosphorylation of cAMP-dependent protein kinase 1 (PKA) substrates and GSK-3α. SIN-1 induced peroxynitrite does not decrease sperm viability, but significantly reduces sperm motility, progressive motility, velocities and motility coefficients. Concomitantly, peroxynitrite does not affect mitochondrial membrane potential, plasma membrane fluidity, or A23187-induced acrosome reaction. However, peroxynitrite significantly increases sperm lipid peroxidation in both media. In conclusion, peroxynitrite compromises boar sperm motility without affecting mitochondrial activity. Although peroxynitrite potentiates the phosphorylation of pathways leading to sperm motility, it also causes oxidative stress that might explain, at least partially, the motility impairment.

## 1. Introduction

Oxidative stress occurs as a result of an imbalance of antioxidant defence mechanisms and free radicals production such as reactive oxygen species (ROS), which also include nitrogen-containing compounds, collectively named reactive nitrogen species (RNS). Low and/or regulated concentrations of cellular RNS (below μM range) play important roles in the physiological function in spermatozoa [1,2,3]. However, when RNS are produced at high concentrations can lead to so-called nitrosative stress that causes adverse effects in mammalian spermatozoa [4,5,6,7,8,9,10], specifically in their function and fertilizing ability [11]. The RNS include peroxynitrite anion (ONOO^−^), nitrogen dioxide, nitroxyl ion, nitrosyl-containing compounds, and nitric oxide (NO). Under physiological conditions, mammalian spermatozoa produce the diffusible free radical NO [1,12] that in appropriate levels contributes to regulate sperm function [2,3,12,13,14]. Nitric oxide can react with superoxide anion (O_2_^●−^), also produced physiologically by spermatozoa, generating peroxynitrite (ONOO^−^) by a very fast and irreversible reaction [13] due to its exothermic nature [15]. In the same way, peroxynitrite can diffuse into the cells and even cross cell membranes and at low concentrations contribute to modulate sperm functions, mainly capacitation, as demonstrated in human spermatozoa [13] or in cryopreserved bovine spermatozoa, where ONOO^−^ might also act as an inducer of sperm capacitation [16]. However, peroxynitrite molecule has been involved in some diseases (reviewed in [17]), because it is a strong oxidizing and/or nitrating agent that covalently binds to a variety of biomolecules causing lipid peroxidation, protein oxidation, nitration of the aromatic-side chains of amino acids as tyrosine and tryptophan, DNA oxidation, and inactivation of different enzymes. Therefore, ONOO^−^ causes oxidative stress that promotes protein damage by direct oxidation of proteins side-chain, but also by adduction of secondary products resulting from the oxidation of polyunsaturated fatty acids, lipid peroxidation [18].

The molecular and functional effects of nitrosative stress induced by high concentrations of ONOO^−^ in spermatozoa have been mainly investigated in the human species [5,7,8,10,13,19], where ROS and RNS promote a dose dependent increase of sperm tyrosine-nitrated proteins and S-glutathionylation and impair sperm motility and capacitation [5,13]. Among negative effects, a positive correlation between RNS and human sperm DNA fragmentation has been demonstrated [20]. Considering specifically ONOO^−^ effects, it has been shown a negative correlation between ONOO^−^ levels and human sperm morphology and motility parameters [9,20]. In fact, Vignini et al., (2006) [9] demonstrated that spermatozoa from normospermic patients have lower peroxynitrite levels, higher values of kinetic parameters and a decrease in tyrosine nitration compared to asthenozoospermic infertile patients. In addition, it had been demonstrated that in vitro exposure of human spermatozoa to ONOO^−^ cause a decrease in total sulfhydryl groups and in motility, in parallel to an increase in lipid peroxidation [21]. Later, the in vitro exposure of human spermatozoa to peroxynitrite has been approached using a donor molecule, 3-morpholinosydnonimine (SIN-1), which effectively generates ONOO^−^ in spermatozoa [7]. Using SIN-1, it has been demonstrated that peroxynitrite-induced nitrosative stress impairs vital functions of human spermatozoa including motility [7], via an alteration of the mitochondrial membrane potential, impairment of sperm ATP production [8], and an increased in thiol groups oxidation of sperm proteins [10], without affecting sperm viability [7]. At longer times, peroxynitrite-mediated nitrosative stress induces a type of cell death known as mitochondrial permeability transition (MPT)-driven necrosis in human spermatozoa [19].

As mentioned before, the majority of studies have evaluated the effect of nitrosative stress induced by ONOO^−^ on spermatozoa from humans. However, no studies have investigated the in vitro effect of peroxynitrite in boar spermatozoa function, as previous works in this species have focused on oxidative stress induced by ROS, especially during semen preservation at 17 °C. Therefore, the aim of this work is to study the in vitro effects of peroxynitrite, generated by the donor molecule SIN-1, on the main functional processes of boar spermatozoa and the intracellular signalling pathways involved.

## 2. Results

### 2.1. Effect of 3-Morpholinosydnonimine (SIN-1) Induced-Peroxynitrite in the Motility of Boar Spermatozoa

**Figure 1:**
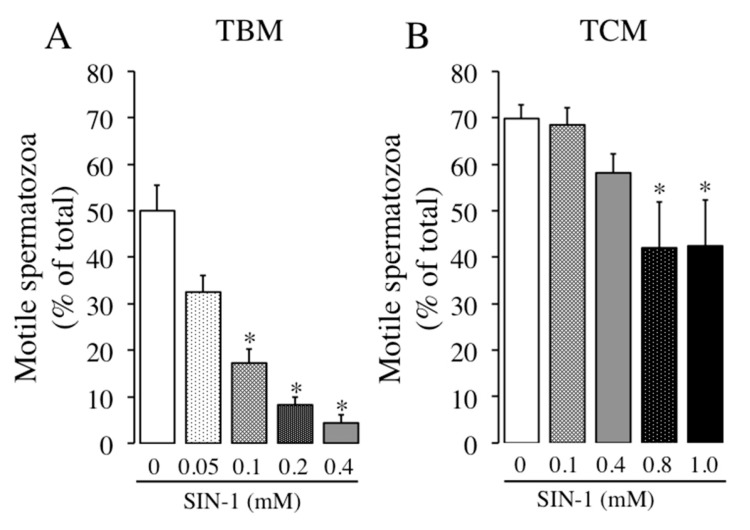
Effects of 3-morpholinosydnonimine (SIN-1) induced-peroxynitrite in the percentage of motile spermatozoa. Boar spermatozoa were incubated in non-capacitating media (TBM) (**A**) or capacitating media (TCM) (**B**) at 38.5 °C in the absence (white histograms) or presence of different concentrations of SIN-1 (0.05-1 mM, filled histograms) for 1 h. The percentage of motile spermatozoa was evaluated by ISAS^®^ system. This experiment was performed 8 times (*n* = 8) and results are expressed as the mean of the percentage of total spermatozoa ± standard error of the mean (SEM). Statistical differences are shown with * (*p* < 0.05).

The in vitro effect of peroxynitrite in boar sperm motility was evaluated using different concentrations of the donor molecule, SIN-1, in both media, non-capacitating (TBM) and capacitating (TCM). As observed in Figure 1, SIN-1 leads to a concentration dependent reduction in the percentage of motile spermatozoa in both media, although there is a clear difference between them as the reduction in sperm motility induced by SIN-1 is stronger in TBM than in TCM at the same SIN-1 concentrations. Thus, whereas the reduction in the number of motile spermatozoa is statistically significant at concentrations of 0.1, 0.2, and 0.4 mM in TBM (Figure 1A), in TCM there is not a visible effect at 0.1 mM and the modest reduction induced by 0.4 mM is not statistically significant (Figure 1B). Therefore, in view of the weaker effect of SIN-1 in TCM we decided to study higher concentrations of SIN-1 in this medium (0.8 and 1 mM). As observed in Figure 1B, these SIN-1 concentrations significantly reduced the percentage of motile spermatozoa but, again, their effect in TCM is weaker than in TBM medium. Thus, the maximal reduction in TBM (92%) occurs at 0.4 mM, where only 4% spermatozoa remain motile, whereas in TCM the maximal reduction in motility is about 40% at 0.8–1 mM, where 42% spermatozoa are motile. Considering these results and for the experiments of this work, we decided to use SIN-1 concentrations of 0.4 mM when incubating in TBM and 0.4 and 1 mM for TCM.

**Figure 2:**
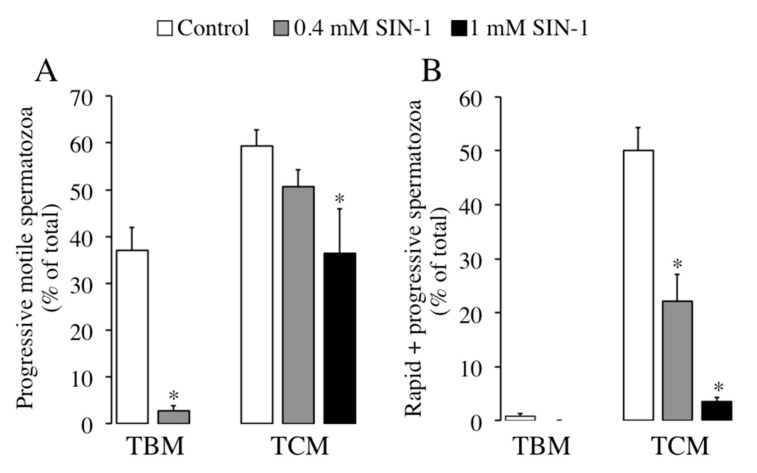
Effects of SIN-1 induced-peroxynitrite in the percentages of progressive motile and rapid + progressive motile boar spermatozoa. Spermatozoa were incubated in TBM or TCM at 38.5 °C in the absence (white histograms) or the presence of indicated concentrations of SIN-1 (filled histograms) for 1 h. Sperm motility parameters were evaluated by ISAS^®^ system: the percentages of motile spermatozoa with progressive motility (**A**) and rapid and progressive motility (**B**) are shown. This experiment was performed 8 times (*n* = 8) and results are expressed as the mean of the percentage of total sperm ± SEM. Statistical differences are shown with * (*p* < 0.05).

The effects of SIN-1 in other motility parameters as the percentages of progressive motile and rapid + progressive motile spermatozoa in both media are shown in Figure 2. Interestingly, SIN-1 treatment leads to a significant reduction in the percentage of progressive motile spermatozoa (Figure 2A) in both TBM (0.4 mM) and in TCM (1 mM). The inhibitory effect of SIN-1 is stronger in TBM (93% reduction, where 0.4 mM reduces the progressive motile spermatozoa to only 3%), than in TCM (39% decrease, where there is 36% of progressive motile spermatozoa). Treatment with SIN-1 in TCM causes a significant reduction (94%) in rapid + progressive motility parameter in a concentration-dependent manner, decreasing to only 3% of rapid + progressive spermatozoa when incubating with 1 mM (Figure 2B). As expected, in TBM medium the rapid + progressive spermatozoa are almost undetectable (0.8%) in absence of SIN-1, making difficult to evaluate the SIN-1 effect under these conditions.

**Figure 3:**
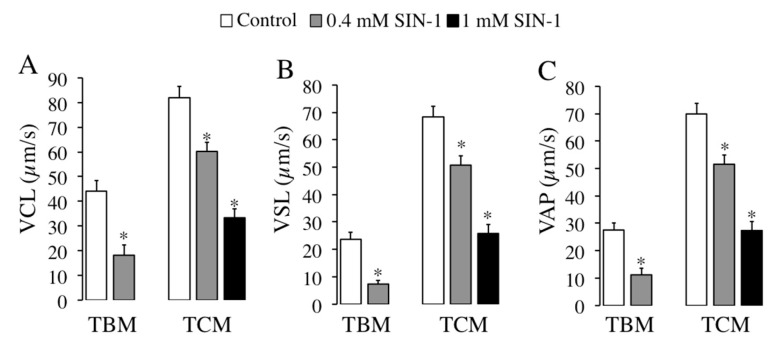
Effects of SIN-1 induced-peroxynitrite in boar spermatozoa velocities. Boar spermatozoa were incubated in TBM or TCM at 38.5 °C in the absence (white histograms) or presence of indicated concentrations of SIN-1 (filled histograms) for 1 h. Boar sperm velocities were evaluated by ISAS^®^ system: curvilinear velocity VCL (**A**), straight-linear velocity VSL (**B**), and average velocity VAP (**C**) expressed as μm/s are shown. This experiment was performed 8 times (*n* = 8) and results are expressed as the mean of the percentage of total sperm ± SEM. Statistical differences are shown with * (*p* < 0.05).

We further evaluated the effect of SIN-1-induced peroxynitrite in other motility parameters evaluated by CASA, such as spermatozoa velocities. As observed in Figure 3, SIN-1 incubation either in TBM or TCM causes a significant and concentration-dependent reduction in any sperm velocity studied: curvilinear VCL (Figure 3A), straight-linear VSL (Figure 3B) or the average VAP (Figure 3C).

This significant effect is detected at 0.4 mM in both media and the highest reductions in sperm velocity are achieved in TBM with 0.4 mM of SIN-1 for VSL (70% reduction) and in TCM with 1 mM for VSL (60% reduction). Moreover, SIN-1 significantly affects others spermatozoa motility coefficients analysed in Table 1. When spermatozoa are incubated with SIN-1 in TBM, the coefficients of straightness (STR) and the beat cross frequency (BCF) are significantly reduced, whereas in TCM, the amplitude of lateral sperm head displacement (ALH) is significantly reduced.

### 2.2. The Treatment with SIN-1 Effectively Generates RNS, as Peroxynitrite, in Boar Spermatozoa

**Figure 4:**
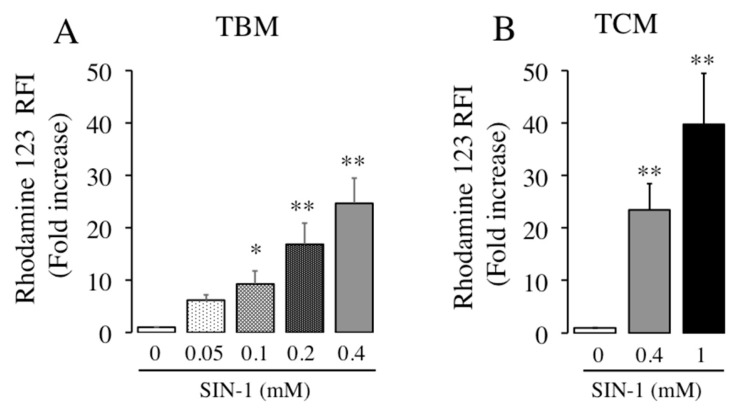
Concentration-dependent generation of peroxynitrite by SIN-1 in boar spermatozoa. Boar spermatozoa were incubated in TBM (**A**) or TCM (**B**) in the absence (white histograms) and presence of indicated concentrations of SIN-1 (filled histograms). The levels of reactive nitrogen species (RNS) induced by SIN-1 were evaluated by flow cytometry using DHR 123 as a probe. These experiments were performed 6 times (*n* = 6) and results are expressed as the fold increase of Relative Fluorescence Intensity (RFI) of DHR 123 ± SEM. Statistical differences are shown with * (*p* < 0.05) and ** (*p* < 0.01).

We next aimed to confirm that the exposure of boar spermatozoa to SIN-1 effectively generates intracellular peroxynitrite, as it is published in human spermatozoa [7]. The generation of peroxynitrite following exposure to SIN-1 was evaluated by flow cytometry using dihydrorhodamine 123 (DHR 123), which becomes highly fluorescent after oxidation to rhodamine 123 by peroxynitrite or any RNS. The treatment of spermatozoa with different SIN-1 concentrations (0.05 to 0.4 mM) during 1 h at 38.5 °C in TBM resulted in a concentration-dependent increase in the relative intensity of fluorescence (RFI) due to oxidized-rhodamine by RNS (Figure 4A), which is statistically significant at SIN-1 concentrations of 0.1, 0.2, and 0.4 mM. In order to compare the effect of SIN-1 as peroxynitrite-inducer in different sperm incubation media, spermatozoa were exposed to SIN-1 in TCM. As observed in Figure 4, SIN-1 (0.4 mM) induces a significant increase in sperm peroxynitrite or RNS amount in both media TBM (Figure 4A) and TCM (Figure 4B). The ability of the same concentration of SIN-1 (0.4 mM) to increase peroxynitrite levels in spermatozoa is similar in both media (24-fold increase). A greater concentration of SIN-1 (1 mM) significantly increases by 40 times the amount of peroxynitrite in boar spermatozoa.

### 2.3. Effect of SIN-1 Induced-Peroxynitrite in the Viability of Boar Spermatozoa

**Figure 5:**
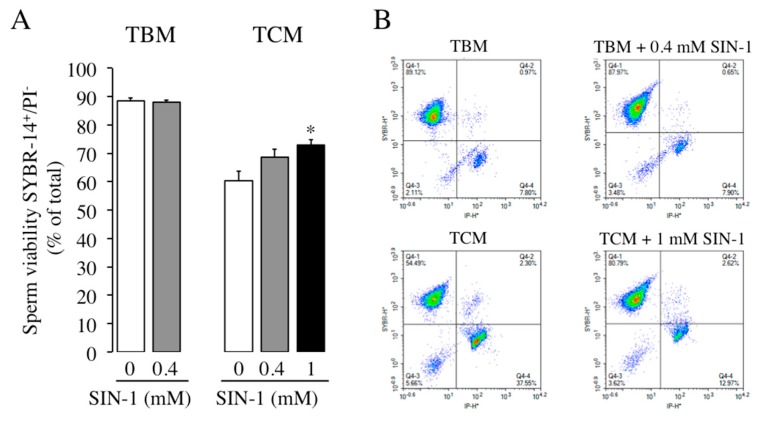
Effect of SIN-1 induced-peroxynitrite in boar spermatozoa viability. (**A**) Boar spermatozoa were incubated in TBM or TCM at 38.5 °C in the absence (white histograms) or presence of SIN-1 (filled histograms) for 1 h. (**B**) Representative two-dimensional SYBR-14 fluorescence versus PI fluorescence dot plots for sperm samples incubated in presence or absence of SIN-1 are shown. This experiment was performed 8 times (*n* = 8) and the results are expressed as the percentage of SYBR14-positive and PI-negative spermatozoa ± SEM. Statistical differences are shown with * (*p* < 0.05).

Sperm viability after 1 h of incubation in control spermatozoa (absence of SIN-1, white histograms) is lower in TCM (60% of total) than in TBM (88% of total) (Figure 5), as previously described [22]. The exposure of spermatozoa to SIN-1 in TBM does not affect the percentage of viable spermatozoa. However, there is a small, although reproducible, effect of SIN-1 increasing sperm viability or preventing the loss of cell viability observed in TCM, where 68% and 73% of spermatozoa remain viable after 0.4 mM and 1 mM of SIN-1, respectively, compared to 60% in the absence of SIN-1 (Figure 5). This modest preventive effect of SIN-1 in the loss of spermatozoa viability occurring in TCM is concentration-dependent and is statistically significant at 1 mM of SIN-1.

### 2.4. Effect of SIN-1 Induced-Peroxynitrite in the Mitochondrial Membrane Potential (ΔΨm) of Boar Spermatozoa

**Figure 6:**
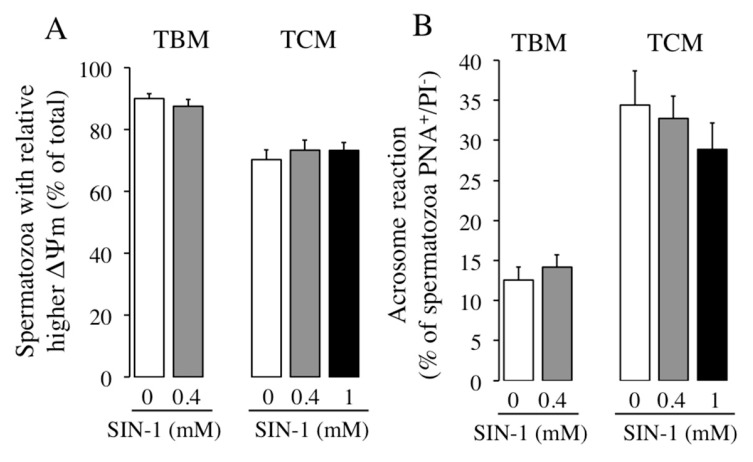
Effect of SIN-1 induced-peroxynitrite in mitochondrial membrane potential and in the acrosome reaction induced by calcium ionophore A23187 on boar spermatozoa. Boar spermatozoa were incubated in TBM or TCM at 38.5 °C in the absence (white histograms) or presence of SIN-1 (filled histograms) for 1 h. (**A**) Results are expressed as the percentage of spermatozoa exhibiting relative higher ΔΨm from the total sperm cells analyzed. (**B**) Percentage of live acrosome-reacted spermatozoa (PNA^+^/PI^−^) induced by A23187. These experiments were performed 8 times in A (*n* = 8) and 7 times in B (*n* = 7) and data are expressed as the mean ± SEM. No statistical differences were found (*p* < 0.05).

The action of peroxynitrite induced by SIN-1 in the sperm mitochondrial membrane potential, ΔΨm, was evaluated after incubation of boar spermatozoa in both media TBM and TCM in the presence or absence of different SIN-1 concentrations. As observed in Figure 6A, an increase in peroxynitrite caused by SIN-1 does not significantly modify the population of boar spermatozoa presenting relative higher ΔΨm at any concentration or incubation medium studied.

### 2.5. Effect of SIN-1 Induced-Peroxynitrite in the Acrosome Reaction Induced by A23187 in Boar Spermatozoa

We analyzed the effect of peroxynitrite generated by SIN-1 on the acrosome reaction of boar spermatozoa induced by calcium ionophore A23187 in TBM and TCM, using PNA-FITC and PI as probes for flow cytometry. As observed in Figure 6B, the percentage of live acrosome-reacted spermatozoa induced by A23187 in a non-capacitating medium is small (12%) and is not significantly affected by SIN-1 treatment. When boar spermatozoa are incubated in TCM, A23187 effectively triggers acrosome reaction (3-fold increase; white histograms) and SIN-1 induced-RNS does not significantly affect the percentage of PNA^+^/PI^−^ spermatozoa, although a slight decrease is detected (grey and black histograms).

### 2.6. Effect of SIN-1 Induced-Peroxynitrite in Lipid Peroxidation Degree and Lipid Organization in the Plasma Membrane of Boar Spermatozoa

**Figure 7:**
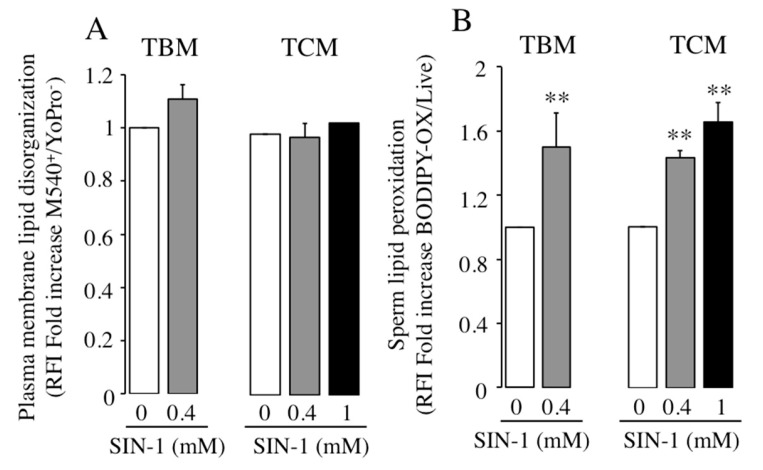
Effect of SIN-1 induced-peroxynitrite in lipid peroxidation and in plasma membrane lipid organization on boar spermatozoa. Boar spermatozoa were incubated in TBM or TCM in the absence (white bars) or presence of indicated concentrations of SIN-1 (filled bars) for 1 h at 38.5 °C. (**A**) Plasma membrane lipid disorganization levels in viable boar spermatozoa (M540^high^/ YoPro-1^−^). (**B**) Lipid peroxidation levels in viable boar spermatozoa (C11-BODIPY^581/591^/L10120^−^). These experiments were performed 6 times (*n* = 6). Results are expressed as the mean ± SEM of fold increase in Relative Fluorescence Intensity (RFI). Statistical differences are shown with ** (*p* < 0.01).

We next investigated whether SIN-1 affects the degree of plasma membrane lipid organization, evaluated by flow cytometry, in boar spermatozoa. Results in Figure 7A show that lipid disorganization of plasma membrane is unaffected by SIN-1-induced peroxynitrite, as the RFI of spermatozoa M540^high^ in living spermatozoa are similar in control and treated spermatozoa. This lack of SIN-1 effect is observed in any sperm incubation medium (Figure 7A).

In order to investigate the effect of SIN-1 induced peroxynitrite in the sperm lipid peroxidation, we used the fluorescent lipid probe C11-BODIPY^581/591^ by flow cytometry. Interestingly, SIN-1-induced peroxynitrite significantly raises lipid peroxidation levels in boar spermatozoa in any incubation medium, TBM or TCM (Figure 7B). In a capacitating medium, SIN-1 treatment increases sperm lipid peroxidation in a concentration-dependent manner.

### 2.7. Effects of SIN-1 Induced-Peroxynitrite in the Signaling Pathways Mediated by cAMP-Dependent Protein Kinase 1 (PKA) and Glycogen Synthase Kinase 3 (GSK-3) in Boar Spermatozoa

**Figure 8:**
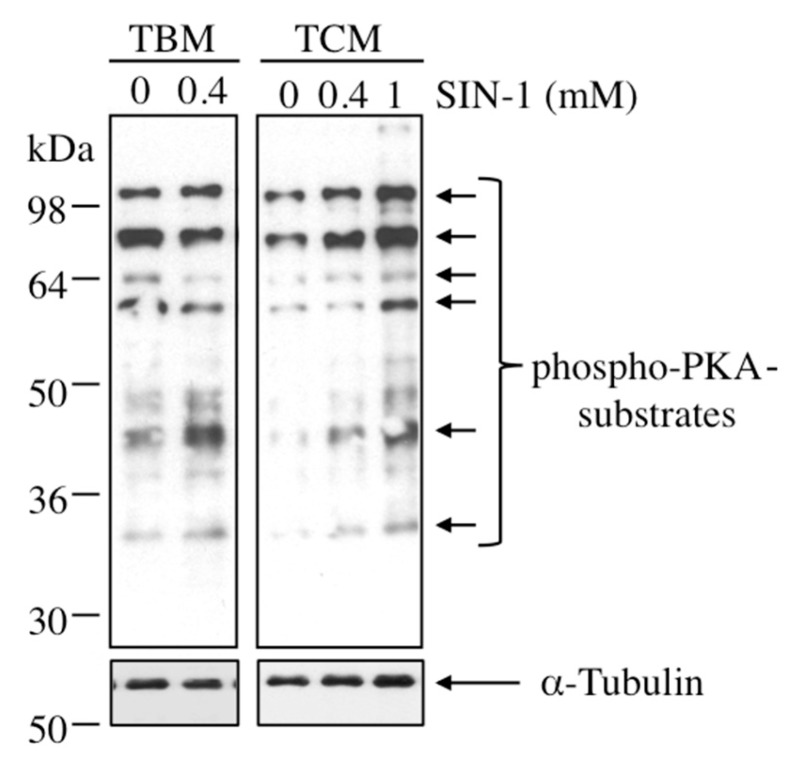
Effect of SIN-1 induced-peroxynitrite in the phosphorylation of PKA-substrates on boar spermatozoa. Spermatozoa were incubated in TBM or TCM medium at the indicated concentrations of SIN-1 at 38.5 °C for 1 h and sperm proteins (15 µg) analyzed by western blotting using anti-phospho-PKA-substrate as primary antibody (upper films). These experiments were performed 4 times and a representative film is shown. Loading controls using anti-α-tubulin antibody (lower films) were performed in a parallel membrane. Arrows indicate the cross-reactive bands of sperm phosphorylated proteins that are substrates of PKA.

We next investigated whether SIN-1-induced peroxynitrite affects those key intracellular signaling pathways mediated by protein phosphorylation that regulate boar sperm motility. As observed in Figure 8, the treatment of boar spermatozoa with SIN-1 modifies the phosphorylation state of the majority of substrates of PKA (indicated by arrows). The increase in the phosphorylation occurs in both media, TBM and TCM, although is more intense in TCM, where a concentration-dependent phosphorylation is detected.

**Figure 9:**
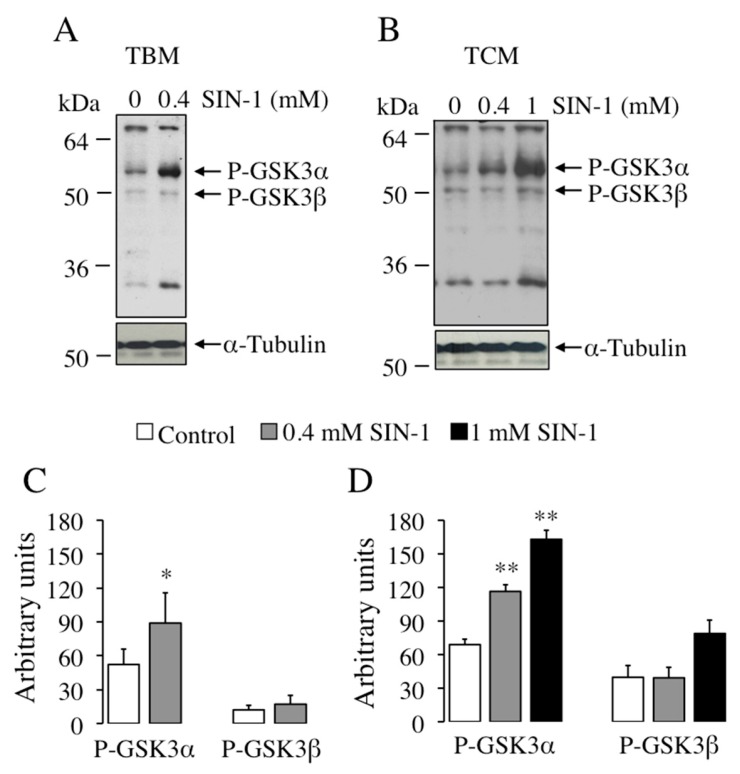
Effect of SIN-1 induced-peroxynitrite in the phosphorylation of GSK-3 on boar spermatozoa. Spermatozoa were incubated in TBM (**A**) or TCM medium (**B**) at the indicated concentrations of SIN-1 at 38.5 °C for 1 h and sperm proteins (15 µg) analyzed by Western blotting using anti-phospho GSK-3α/β as primary antibody (upper films). These experiments were performed 4 times and representative films are shown. Loading controls using anti-α-tubulin antibody (lower films) were performed for each experiment in the same membrane. (**C**,**D**) correspond to the quantification of bands in (**A,****B**), respectively. Arrows indicate the cross-reactive sperm bands corresponding to phosphorylated GSK-3α (P-GSK-3α) and GSK-3β (P-GSK-3β) and also α-tubulin. Densitometry analysis of each band is shown at the bottom image, values are expressed as arbitrary units; mean ± SEM. Statistical differences are shown with ** (*p* < 0.01) and * (*p* < 0.05).

Regarding GSK-3, SIN-1-induced peroxynitrite leads to a clear increase in the phosphorylation state of the α isoform of this kinase (Figure 9). As occurs with the PKA substrates (Figure 8), the increase in phosphorylation of GSK-3α is more pronounced in TCM medium (Figure 9B), where is also observed a concentration-dependent rise: 1.7 times at 0.4 mM and 2.4 times at 1 mM. This GSK-3α phosphorylation effect is statistically significant at both SIN-1 concentrations compared to untreated control.

## 3. Discussion

This study demonstrates that the generation of reactive nitrogen species (RNS), peroxynitrite, following exposure to the donor SIN-1 effectively occurs in boar spermatozoa, as it happens in human spermatozoa [7]. The increase in the intracellular ONOO^−^ amount in boar spermatozoa is dependent of the SIN-1 concentration used (0.05 to 1 mM), which also agrees with the previous work in human spermatozoa [7]. Peroxynitrite generation and its effect in boar spermatozoa are also dependent on the incubation medium, which is supported by the study of Crow [23] showing that ONOO^−^ cellular effects are dependent on the environment in which the anion is present. Interestingly, boar spermatozoa are more sensitive to SIN-1-induced ONOO^−^ than human spermatozoa, as the inhibitory effect of sperm motility with the same SIN-1 concentration is greater in boar, even with only 1 h of incubation versus 4 h in human [7]. This suggests that sensitivity of spermatozoa to RNS, as peroxynitrite, varies with the species studied.

This study shows that SIN-1-induced ONOO^−^ causes a clear reduction on total, rapid and progressive motility in boar spermatozoa, as well as on main sperm kinetic parameters. This inhibition of sperm motility is dependent of the ONOO^−^ concentration and occurs in both sperm incubation media, although is clearly more intense in TBM (92% reduction with 0.4 mM SIN-1) than in TCM (17% reduction with 0.4 mM SIN-1). These data support the previous idea that peroxynitrite effects are dependent on the environment [23], and indicate that ONOO^−^ effects in boar spermatozoa are effectively influenced by the composition of extracellular medium, as the exposition to calcium, bicarbonate or BSA (TCM) clearly prevents the adverse ONOO^−^ effect in sperm motility observed in the absence of these stimuli (TBM). In fact, BSA has been pointed as a protective factor of boar sperm quality during liquid storage at 17 °C, acting as an antioxidant [24]. Moreover, at physiological temperature, BSA is also able to modulate boar sperm motility effect induced by the AMPK activator A769662 [22]. In this work, BSA is not playing an antioxidant role, as the level of sperm lipid peroxidation is the same in presence or absence of BSA, but it could be reasonable to point to BSA as a stimulus that might be modulating the adverse ONNO- effect in sperm motility. Future experiments are needed to clarify this particular issue.

The fact that SIN-1-induced ONOO^−^ inhibits boar spermatozoa progressive motility and main kinetic parameters is in total agreement with studies performed with SIN-1 in human spermatozoa by Uribe et al., [7], which conclude that ONOO^−^ impairs spermatozoa essential functions, as motility. Previously, Öztezcan et al., [21] demonstrated that exogenous peroxynitrite added to human spermatozoa decreased motility and sperm velocities. Later, Vignini et al., [9] showed that endogenous ONOO^−^ produced physiologically in human spermatozoa negatively affected motility, as spermatozoa from asthenozoospermic infertile patients have higher ONOO^−^ levels and lower values of kinetic parameters compared to normospermic fertile patients. However, one study reported that the percentage of human motile spermatozoa was unaffected by peroxynitrite or SIN-1 and that the sperm velocity VCL increased [13]. Different effects of SIN-1 on sperm motility between studies can be attributed to the distinct species studied and also to the incubation times used: 1 h in boar, 4 h in human [7], or 8 h in human [13], as ONOO^−^ amount in spermatozoa generated by SIN-1 greatly depends on the incubation time [7].

A reduction in sperm motility could be explained by sperm death. Interestingly, in the absence of SIN-1, boar sperm viability is lower in TCM than in TBM, which agree with previous results showing that sperm viability decreases in TCM in a time-dependent manner [22]. In the presence of SIN-1, the reduction in sperm motility cannot be attributed to a death of sperm cells, as boar sperm viability is not decreased by SIN-1, which agrees with previous reports in cryopreserved bovine spermatozoa [16] and also in human spermatozoa treated with SIN-1 for longer incubation time, 4 h [7]. In addition, our data focused at the sperm plasma membrane level, reveal that ONOO^−^ does not cause any visible effect in its fluidity, indicating that, at least in our conditions, the impairment in boar sperm motility induced by SIN-1 cannot be attributed to lipid disorganization at the plasma membrane. There are not previous studies investigating SIN-1 effect in this sperm parameter.

One possible explanation for the impairment of boar sperm motility is based on the fact that ONOO^−^ could alter the mitochondrial activity [7]. However, this is not the case in boar spermatozoa, as the mitochondrial membrane potential (ΔΨm) is unaffected by SIN-1 in our conditions. By contrast, in human spermatozoa SIN-1 concentration-dependently decreased the ΔΨm after 1 h [7], and inhibits sperm ATP production after 4 h by affecting metabolic pathways of mitochondrial OXPHOS and glycolysis [8]. Different results about sperm ΔΨm could be attributed to the different species studied (boar or human) because, as mentioned before, there is a species-specific sensitivity to SIN-1 and also to the incubation time with the ONOO^−^ donor, 1 h versus 4 h [8]. At longer incubation times, 24 h, SIN-1 induced-nitrosative stress causes human sperm death due to an induction of mitochondrial permeability transition-driven necrosis [19].

This study suggests that SIN-1-induced ONOO^−^ does not affect boar sperm capacitation, evaluated as the sperm response to acrosome reaction induced by calcium ionophore. Both sperm processes are modulated by NO [25] and it has been proposed that ONOO^−^ also might modulate sperm capacitation [26], as reported in human spermatozoa [13] and in cryopreserved bovine spermatozoa [16], where SIN-1 increased the sperm acrosome reaction induced by different stimuli. Specifically, in boar spermatozoa there are discrepancies regarding the involvement of NO pathway in the acrosome reaction. Thus, Staicu et al., (2019) [27] recently found that exogenous NO does not affect acrosome reaction, whereas Hou et al., (2008) [28] described that NO donor increases the number of acrosome-reacted spermatozoa. Our results with ONOO- support the idea that the pathway of NO, including related RNS, is not likely involved in the acrosome reaction of boar spermatozoa.

Peroxynitrite, as a strong oxidant, causes oxidative stress that promotes protein damage by direct oxidation and by adduction of secondary products resulting from the oxidation of polyunsaturated fatty acids [18,29]. In fact, in this study, sperm peroxynitrite generated after 1 h of incubation with SIN-1 causes a marked increase in lipid peroxidation in boar spermatozoa. A previous study points to an increase in lipid peroxidation due to ONOO^−^ as the cause of human sperm dysfunction [21]. Thus, we propose that the motility impairment in boar spermatozoa induced by SIN-1 can be attributed, at least in part, to an oxidative stress induced by peroxynitrite. This conclusion is supported by previous studies in human spermatozoa reporting that oxidative stress impairs human spermatozoa function [5,30,31] and that lipid peroxidation effectively causes loss of human sperm motility [32]. A consequence derived from oxidative stress is that ONOO^−^ rapidly reacts with the sulfhydryl group, as reported in human spermatozoa, where the adverse effect of peroxynitrite in motility is mediated by an increased oxidation in the thiol groups of sperm proteins [10] or by depletion of total sulphydryl group in sperm proteins [21]. In addition, ONOO^−^ can cause tyrosine nitration, mostly in specific functional domains of sperm proteins, promoting structure and conformations changes that may be responsible for the alteration or inactivation of sperm proteins function [13,33]. Although we have not specifically evaluated protein tyrosine nitration or thiol oxidation groups in boar spermatozoa, we demonstrate that lipid peroxidation occurs in response to ONOO^−^ and thus, we propose that those sperm proteins very sensitive to oxidative stress that are involved in molecular mechanisms of sperm motility can likely become oxidized and inactivated by ONOO^−^, leading to an impairment in boar sperm motility.

Among other actions, peroxynitrite might function as cellular messenger [13,26] and therefore this RNS might regulate sperm function through the modulation of intracellular signaling pathways, as it has been reported in human spermatozoa [34]. To our knowledge, this is the first study that demonstrates that SIN-1-induced ONOO^−^ acts as a modulator of serine/threonine phosphorylation in boar spermatozoa. In fact, peroxynitrite promotes an increase in the phosphorylation of GSK-3α and in sperm proteins that are substrates of PKA. Both signaling pathways, GSK-3α and PKA, are essential in the regulation of boar sperm motility [35,36,37]. Part of our results with ONOO^−^ are in line with a recent study in boar spermatozoa that found that phosphorylation of PKA substrates is significantly lower when using NO synthase inhibitors [27]. The peroxynitrite-induced increase in GSK-3α phosphorylation is greater in TCM (2.4 fold-increase over control), than in TBM (1.7 fold-increase) as stimulating medium TCM alone is already promoting higher levels of phosphorylated GSK-3α than the un-stimulating TBM. Thus, according to its regulatory role in motility [35,36], the greater GSK-3α phosphorylation induced by ONOO^−^ would explain the greater percentage of sperm motility evaluated in TCM, where 40% of total spermatozoa still remain motile (0.8–1 mM SIN-1; Figure 1B). By contrast, the lower fold increase in GSK-3α phosphorylation caused by peroxynitrite in TBM would account for much lower sperm motility under TBM, where only 4% of total spermatozoa are motile (0.4 mM SIN-1; Figure 1B). At the molecular level, the peroxynitrite-induced phosphorylation of PKA and GSK-3α in boar spermatozoa can be explained by the fact that ONOO^−^, besides to stimulate guanylate cyclase and therefore increasing cGMP levels, can also increase cAMP levels when they are already stimulated by low concentrations of adenylate cyclase (AC) activators, as it has been demonstrated in platelets in response to ONOO^−^ [38], and to NO donors [39]. Moreover, similar synergistic peroxynitrite effect has been described also in vascular smooth muscle cells when AC becomes activated with forskoline or sodium fluoride [40]. However, the elevating effect of ONOO^−^ in the cAMP levels is not likely due to a direct action on the AC, but rather to an inhibition of phosphodiesterases, as found for phosphodiesterase type III in human platelets [38]. Based on these evidences in somatic cells, we can postulate that in boar spermatozoa also a synergistic effect between ONOO^−^ and soluble AC (sAC) activators might be leading to an increase in sperm cAMP levels that could explain the increase observed in the phosphorylation of its downstream pathways, i.e., PKA substrates and GSK-3α, mainly in TCM medium that includes sAC activators. This molecular mechanism could also contribute to explain the small but significant increase in boar sperm viability due to ONOO^−^ in TCM, where sperm sAC is activated, as it has been demonstrated that somatic cells, such as monocytes and macrophages, survive to ONOO^−^ via a concomitant activation of PKA pathway [41]. Surprisingly, ONOO^−^ raises an issue in boar spermatozoa: it leads to phosphorylation of two key signaling pathways involved in sperm motility but ultimately it inhibits sperm motility. We think that future investigation is needed to elucidate the exact molecular mechanisms triggered by ONOO^−^ that lead to a reduction of sperm motility, but the most plausible explanation based in our results is that sperm oxidative stress is the ultimate cause of inactivation of proteins involved in sperm motility, independently of their phosphorylation state.

In conclusion, peroxynitrite compromises a vital function of the male gamete, the sperm motility, without affecting the mitochondrial membrane potential. Although peroxynitrite potentiates the phosphorylation of two serine/threonine kinases, PKA and GSK-3α, leading to sperm motility, it also causes oxidative stress that might contribute, at least partially, to explain the sperm motility impairment.

## 4. Materials and Methods

### 4.1. Reagents and Sources

3-Morpholinosydnonimine (SIN-1) and dihydrorhodamine 123 (DHR 123) probe were from ENZO Life Science Inc. (Farmingdate, NY, USA); Propidium iodide (PI), SYBR-14, and M540 and YoPro-1 probes were purchased from Molecular Probes (Leiden, The Netherlands); PNA-FITC and calcium ionophore A23187 were from Sigma-Aldrich (St Louis, MO, USA); C11-BODIPY 581/591 (4,4-difluoro-5- (4-phenyl-1,3-butadienyl)-4- bora-3a, 4a-diaza-s-indacene-3-undecanoic acid)and JC-1 probes from Life Technologies Ltd (Grand Island, NY, USA); coulter isotone II diluent from Beckman Coulter Inc. (Brea, CA, USA); *DC* ™ Protein Assays and 2× Laemmli Sample Buffer from Bio-Rad (Hercules, CA, USA). ECL detection kit and LIVE/DEAD™ Fixable Far Red Dead Cell Stain Kit (L10120) were from Thermo Scientific (Rockford, USA). Furthermore, the anti-phospho (Ser/Thr) PKA Substrate (#9624) and anti-phospho (Ser21/9) GSK3α/β (#9331) polyclonal antibodies were from Cell Signaling Technology, Inc. (Beverly, MA, USA); the anti-α-tubuline antibody (TU-02, #SC-8035) was from Santa Cruz Biotechnology (Santa Cruz, CA, USA). All reagents used to prepare incubation media were purchased from Sigma-Aldrich (St. Louis, MO, USA).

### 4.2. Spermatozoa Incubation Media

Tyrode’s basal medium (TBM; 96 mM NaCl, 4.7 mM KCl, 0.4 mM MgSO_4_, 0.3 mM NaH_2_PO_4_, 5.5 mM glucose, 1 mM sodium pyruvate, 21.6 mM sodium lactate, 20 mM HEPES, 5 mM EGTA, and 0.02% PVA) was prepared and used as the non-capacitating medium. A variant of TBM was made omitting EGTA and adding 1 mM CaCl_2_, 15 mM NaHCO_3_ and 3 mg/mL BSA, then it was equilibrated with 5% CO_2_ in O_2_ and termed Tyrode’s complete medium (TCM), a spermatozoa-capacitating medium. All media were prepared on the day of use and adjusted to pH 7.45 with an osmolarity of 290–310 mOsm kg^−1^.

### 4.3. Boar Semen Collection and Experimental Treatment of Spermatozoa with 3-Morpholinosydnonimine (SIN-1)

Sperm samples from Duroc boars (2–4 years old) were commercially obtained from a regional porcine company (Tecnogenext, S.L, Mérida, Spain), without any requirement of approval from the animal research review board of the University of Extremadura. All boars were housed in individual pens in an environmentally controlled building (15–25 °C) according to Regional Government and European regulations, and received the same diet. Fresh ejaculates were collected with the gloved hand technique and stored at 17 °C before use in the laboratory. In order to minimize individual boar variations, samples from up to 3 animals were pooled using semen from no less than 12 boars in different combinations. Only semen pools with at least 80% morphologically normal spermatozoa were used. Semen was centrifuged at 900 *g* for 4 min, washed with phosphate-buffered saline (PBS) and spermatozoa were placed in TBM or TCM medium to a final concentration of 40 × 10^6^ spermatozoa/mL.

Depending on each experimental procedure, different volumes of spermatozoa samples (0.5 or 1.5 mL) containing 40 × 10^6^ spermatozoa/mL were incubated in the presence or absence of 3-morpholinosydnonimine (SIN-1) at 38.5 °C for 1 h. When spermatozoa were incubated in TBM, incubation was performed in absence of CO_2_, whereas TCM treatment was performed in a humidified atmosphere of 5% CO_2_. To confirm that SIN-1 is effectively a peroxynitrite donor in boar spermatozoa, samples were initially treated with or without different concentrations of SIN-1: 0.05, 0.1, 0.2, 0.4, and 1 mM. As a control sample, spermatozoa were incubated with the vehicle dimethyl sulfoxide (DMSO) at the highest concentration used (0.4% in experiments including TBM and 1% in experiments under TCM).

### 4.4. Flow Cytometry Analysis

Flow cytometry analysis was performed using an ACEA NovoCyte^®^ flow cytometer (ACEA Biosciences, Inc., San Diego, CA, USA) equipped with a three detection channels for blue laser (488 nm): BL-1 (530 ± 30 nm band pass filter); BL-2 (572 ± 28 nm band pass filter) and BL-4 (675 ± 30 nm band pass filter) and a detection channel for a red laser (640 nm): BL-3 (660 ± 20 nm band pass filter). Flow cytometry experiments and data analyses were performed using ACEA Novo Express^®^ software (ACEA Biosciences, Inc., San Diego, CA, USA). Fluorescence data were represented in a logarithmic scale.

### 4.5. Generation and Detection of Peroxynitrite in Boar Spermatozoa by Flow Cytometry

SIN-1 was used for the generation of intracellular peroxynitrite, as it has been previously described in human spermatozoa [7]. A stock solution of SIN-1 was prepared at 100 mM in DMSO. Briefly, aliquots of 0.5 mL boar sperm samples (40 × 10^6^ spermatozoa/mL) were incubated with different concentrations of SIN-1 or DMSO at 38.5 °C for 1 h. After incubation, sperm samples were incubated with 1 μM of the fluorescent probe dihydrorhodamine 123 (DHR 123) at 37 °C for 20 min to detect peroxynitrite production in boar spermatozoa. DHR 123 is oxidized by peroxynitrite into rhodamine 123 and not by other oxidants such as H_2_O_2_, superoxide anion O_2_^●−^ or NO [42]. After excitation at 488 nm, rhodamine 123 fluorescence was detected using a 530 ± 30 nm band pass filter. Results are expressed as the relative fluorescent intensity (RFI) normalized to the control ± SEM; the fluorescence values were calculated on the geometric mean fluorescence intensity (MFI) of rhodamine 123. We used RFI because it allows a better estimation of progressive changes in the whole cell population.

### 4.6. Analysis of Spermatozoa Viability by Flow Cytometry

As described previously [22,43], fluorescent staining using SYBR-14 and propidium iodide (PI) was performed to measure sperm viability. Briefly, 5 μL of SYBR-14 (2 μM) and 10 μL of PI (240 µM) were added to 100 µL of spermatozoa (40 × 10^6^ cells/mL) diluted with 400 µL of isotonic buffered diluent, then incubated for 15 min at room temperature (RT) in darkness and analyzed in the flow cytometer. After excitation at 488 nm, SYBR-14 fluorescence was detected using a 530 ± 30 nm band pass filter and PI fluorescence using 675 ± 30 nm band pass filter. Results of viable spermatozoa were expressed as the average of the percentage of SYBR14^+^ and PI^−^ spermatozoa ± SEM.

### 4.7. Evaluation of the Sperm Acrosome Membrane Integrity by Flow Cytometry

Acrosome integrity was assessed using PNA-FITC as described previously [43]. Aliquots of 100 µL of semen (40 × 10^6^ cells/mL) were incubated (darkness, 5 min at RT) with 2 µg/mL of PNA-FITC and 6 µM of PI. Then, 400 µL of isotonic buffered diluent were added and mixed before flow cytometry analysis. The fluorescence value of probe PNA-FITC was collected in the BL-1 channel and in the BL-4 channel for PI. Results are expressed as the average of the percentage of PNA^+^ and PI^−^ spermatozoa ± SEM.

### 4.8. Analysis of Mitochondrial Membrane Potential (ΔΨm) of Boar Spermatozoa by Flow Cytometry

Variations of mitochondrial membrane potential in boar spermatozoa, ΔΨm, were evaluated using the specific probe JC-1 (5,5′,6,6′–tetrachloro-1,1′,3,3′ tetraethylbenzymidazolyl carbocyanine iodine) as described previously [43,44]. Briefly, 100 µL of spermatozoa (40 × 10^6^ cells/mL) were diluted in 400 µL of isotonic buffered diluent containing 0.9 µM of JC-1 and incubated at 38.5 °C for 30 min. The fluorescence value of JC-1 was collected on both channels BL-1 and BL-2. The percentage of orange stained sperm cells was recorded and considered the population of spermatozoa with a relative higher mitochondrial membrane potential. Results are expressed as the average of the percentage of orange stained (high ΔΨm) spermatozoa ± SEM.

### 4.9. Analysis of Lipid Peroxidation in Boar Spermatozoa by Flow Cytometry

Lipid peroxidation (LPO) was assessed using the fluorescent probe C11-BODIPY^581/591^, which becomes oxidized in contact with reactive species (ROS), as described by Brouwers and Gadella [45] with some modifications. Fluorescence emission of the probe changes according to its state (non peroxidized: red and peroxidized: green), and was detected by flow cytometry. Briefly, sperm samples (40 × 10^6^ cells/mL) were incubated with 2 μM of C11-BODIPY^581/591^ for 30 min at 38.5 °C in TBM. Then, excess of probe C11-BODIPY^581/591^ from the medium was removed by centrifugation and experimental treatment of spermatozoa with SIN-1 was carried out. Later, samples were incubated for 30 min at 38 °C in darkness with LIVE/DEAD™ L10120 kit (amine reactive dye) following the manufacturer’s instructions. Incubation of spermatozoa with 800 μM of FeSO_4_ as an oxidant inducer served as control sample for lipid peroxidation. The percentage of viable (L10120^−^) spermatozoa with low fluorescence intensity for C11-BODIPY^581/591^ was recorded. The fluorescence values of peroxidized C11-BODIPY^581/591^ were collected in the BL-1 channel, whereas live/dead cells fluorescence was collected in the BL-3 channel. Results are expressed as the mean of RFI of oxidized C11-BODIPY^581/591^ ± SEM in live spermatozoa.

### 4.10. Analysis of Plasma Membrane Fluidity in Boar Spermatozoa by Flow Cytometry

Fluorescent staining using the probes merocyanine M540, as a membrane lipid fluidity marker, and YoPro-1, as a marker of changes in plasma membrane permeability (commonly associated with cell death), was performed as we previously described [46,47]. Briefly, 100 µL of spermatozoa (40 × 10^6^ cells/mL) were diluted in 400 µL of isotonic buffer containing 75 nM of YoPro-1, mixed and incubated at 38 °C for 15 min. Then, M540 was added to each sample to a final concentration of 2 µM, incubated for 2 min and remixed before flow cytometry analysis. The fluorescence values of probes YoPro-1 and M540 were collected on both BL-1 and BL-2 channels. Labeled spermatozoa were categorized as (i) viable cells with low plasma membrane lipid disorder (YoPro-1^−^/M540^low^), (ii) viable cells with high plasma membrane lipid disorder (YoPro-1^−^/M540^high^); or (iii) non-viable cells (Yo-Pro-1^+^). Results are expressed as the mean of RFI of M540^high^ ± SEM in live spermatozoa (Yo-Pro-1^−^).

### 4.11. Evaluation of Boar Spermatozoa Motility

Boar spermatozoa were incubated with SIN-1 or DMSO at 38.5 °C in TBM or TCM during 1 h. After gentle mixing 2 µL of sperm sample were placed in a 38.5 °C pre-warmed counting chamber with 20 μm depth (Leja^®^, Luzernestraat, The Netherlands) and motility parameters were measured using a microscope equipped with a 10× negative-phase contrast objective and a heated stage. Analysis was based on the examination of 25 consecutive digitalized images obtained during 1 s from at least 4 different fields. At least 300 spermatozoa per sample were analyzed. Finally, a CASA system (ISAS^®^ program, Proiser R+D, Paterna, Valencia, Spain) was used to analyze following sperm motility parameters and coefficients: motile spermatozoa (percentage of spermatozoa with an average path velocity > 10 µm/s), progressive motile spermatozoa (percentage of spermatozoa with a straightness coefficient > 80%), rapidly progressive spermatozoa (percentage of spermatozoa with VAP velocity > 45 μm/s and STR > 80%), curvilinear velocity in µm/s (VCL), straight-line velocity in µm/s (VSL), average path velocity in µm/s (VAP), linearity coefficient in % (LIN), straightness coefficient in % (STR), amplitude of lateral head movement in µm (ALH), beat cross of flagellum frequency (BCF), and wobble coefficient in % (WOB).

### 4.12. Analysis of Boar Spermatozoa Phosphorylated Proteins by Western Blotting

Western blot analysis was performed as previously described [48]. Briefly, spermatozoa (1.5 mL) were centrifuged at 5000 *g* for 3 min at RT and washed in PBS. Pellet was resuspended in 90 μL of Laemmli Sample Buffer (2×) and then centrifuged at 10.000 *g* for 10 min at 4 °C and the protein concentration of the supernatant was determined using a Bio-Rad DC Protein Assay. After protein concentration analysis, 2-mercaptoethanol (2.5%; *v*/*v*) was added to the sperm lysates before heating for 5 min at 95 °C. Sperm proteins (15 μg) were resolved using 10% SDS-PAGE and electro-transferred to nitrocellulose membranes. Membranes were incubated at 4 °C overnight using anti-phospho-GSK-3α/β (1:1.000), anti-phospho-PKA-substrates (1:1.000), or anti-α-tubulin (1:5.000) antibodies. Incubations with the appropriate secondary antibody were used and protein bands on the membrane were visualized using ECL detection kit and exposed to Hyperfilm^TM^ ECL. Individual bands intensity was quantified by densitometry on a gel documentation system using the ImageJ program.

### 4.13. Statistical Analysis

The effects of treatment with SIN-1 at different concentrations were analyzed. In order to show if the differences are statistically significant, hypothesis tests were carried out. Data were analyzed for normal distribution with a Kolmogorov-Smirnov test and for homoscedasticity with a Levene test. When possible, parametric test, as unpaired Students *t*-test or one-way analysis of variance (ANOVA) followed by post-hoc Tukey, were applied (Data in Figure 1, Figure 2, Figure 3, Figure 4, Figure 5, Figure 6 and Table 1). When parametric tests could not be applied, since the data did not meet the applicability conditions (normality and heterocedasticity), nonparametric alternatives were used. Specifically, Kruskal–Wallis test was used to show statistically significant differences among all the groups. When significant results were found, nonparametric pairwise comparisons were used based on the Mann-Whitney U test with the Bonferroni correction (Data in Figure 7 and Figure 9). All data are shown as the mean ± Standard Error of the Mean (SEM). All analyses were performed using SPSS v19 for Windows software (SPSS Inc. Chicago, IL). Statistical significances were set at *p* < 0.05.

## Figures and Tables

**Table 1:** Effects of SIN-1 induced-peroxynitrite in boar spermatozoa motility coefficients.

Treatment	LIN (%)	STR (%)	WOB (%)	ALH (µm)	BCF (Hz)
TBM	58.30 ± 3.31	82.88 ± 1.98	67.15 ± 2.91	1.86 ± 0.14	6.82 ± 0.61
+ 0.4mM SIN-1	52.17 ± 5.90	70.49 ± 4.41 *	69.26 ± 4.04	1.18 ± 0.26	2.02 ± 0.40 *
TCM	78.86 ± 2.00	90.88 ± 1.25	83.49 ± 1.25	2.24 ± 0.10	7.54 ± 0.78
+ 0.4 mM SIN-1	80.66 ± 1.64	91.94 ± 0.90	84.81 ± 1.07	1.71 ± 0.07 *	8.35 ± 0.25
+ 1.0 mM SIN-1	75.48 ± 3.06	88.02 ± 1.68	83.14 ± 2.00	1.36 ± 0.08 *	5.50 ± 0.66

Boar spermatozoa were incubated in TBM or TCM at 38.5 °C in the absence or presence of indicated concentrations of SIN-1 (mM) for 1 h. Sperm kinematic parameters were evaluated by ISAS^®^ system: linearity (LIN, in %); straightness (STR, in %); wobble movement coefficient (WOB, in %); beat cross frequency (BCF, in Hz) and amplitude of lateral sperm head displacement (ALH, in µm). This experiment was performed 8 times (*n* = 8) and values are expressed as the mean ± SEM. Statistical differences from their own control are shown with * (*p* < 0.05).
